# Preliminary Quantitative MRI Assessment After Combined Posterior Endoscopic Cervical Discectomy and Foraminotomy: An Exploratory Retrospective Cohort Study

**DOI:** 10.3390/jcm15114129

**Published:** 2026-05-27

**Authors:** Tomasz Sienkiel, Barbara Jasiewicz, Dominik Taterra, Marcin Gąska, Przemysław Koszyk, Klemens Machajewski, Artur Gądek

**Affiliations:** 1 Department of Orthopedics, University Orthopedic and Rehabilitation Hospital, 34-500 Zakopane, Poland; basiajasiewicz@gmail.com (B.J.); dominiktaterra@uj.edu.pl (D.T.); marcin_gaska@vp.pl (M.G.); przemyslawkoszyk@gmail.com (P.K.); 2 Department of Orthopedics, Medical College, Jagiellonian University, 31-008 Kraków, Poland; arturgadek@uj.edu.pl; 3 Department of Orthopedics and Traumatology, University Hospital in Kraków, 30-688 Kraków, Poland; klemensmachajewski@gmail.com

**Keywords:** posterior endoscopic cervical foraminotomy, cervical radiculopathy, endoscopic cervical discectomy, minimally invasive spine surgery, quantitative MRI, foraminal stenosis, exploratory study, motion-preserving surgery

## Abstract

**Background/Objectives**: Posterior endoscopic cervical foraminotomy is an established motion-preserving procedure for selected patients with unilateral cervical radiculopathy. However, isolated foraminal decompression may be insufficient in cases with concomitant foraminal stenosis and lateral soft disk herniation. This preliminary study evaluated clinical outcomes and quantitative MRI changes after combined posterior endoscopic cervical diskectomy and foraminotomy (CEDF) and explored the relationship between postoperative foraminal enlargement and clinical improvement. **Methods**: This retrospective single-center exploratory cohort study included 15 consecutive patients with single-level unilateral cervical radiculopathy caused by combined foraminal stenosis and lateral soft disc herniation who were treated between 2021 and 2023. All patients underwent CEDF using a posterior full-endoscopic approach. Clinical outcomes were assessed preoperatively, at 6 weeks, and at 12 months using the Visual Analog Scale for arm and neck pain, the Neck Disability Index, and modified MacNab criteria. Quantitative MRI assessment included minimal foraminal diameter, Foraminal Symmetry Index (FSI), and Quantitative Cervical Expansion (QCE). Correlations between radiological and clinical outcomes were analyzed as exploratory, hypothesis-generating analyses. **Results**: Mean minimal foraminal diameter increased from 1.9 ± 0.7 mm preoperatively to 4.1 ± 0.8 mm postoperatively, with improvement in FSI from 0.40 ± 0.12 to 0.89 ± 0.11. Significant clinical improvement was observed across all outcome measures. Mean arm pain decreased from 7.2 ± 1.3 preoperatively to 1.3 ± 1.4 at final follow-up, while NDI improved from 48.0 ± 14.0% to 18.3 ± 12.0%. The minimum clinically important difference for arm pain reduction was achieved in 14 of 15 patients. A moderate positive exploratory association was observed between foraminal enlargement and reduction in arm pain severity. No major neurological complications, postoperative instability, or revision procedures were observed in this small cohort during the available follow-up. **Conclusions**: In this preliminary retrospective single-center cohort, CEDF was associated with clinical improvement and measurable postoperative foraminal enlargement in carefully selected patients with unilateral cervical radiculopathy caused by combined foraminal stenosis and lateral soft disc herniation. The observed association between foraminal enlargement and arm pain reduction should be interpreted cautiously because of the small sample size and exploratory design. QCE and FSI should be regarded as preliminary quantitative radiological indices rather than validated markers of decompression adequacy or clinical response. Larger prospective comparative studies are required to validate these findings and define the role of CEDF among established cervical decompression procedures.

## 1. Introduction

Cervical radiculopathy is a common cause of neck and upper-extremity pain resulting from compression or irritation of the cervical nerve roots. The most frequent etiologies include foraminal stenosis, lateral or foraminal disk herniation, uncovertebral hypertrophy, and degenerative facet joint changes. These pathologies may lead to radiating arm pain, sensory disturbances, motor weakness, and functional impairment. The primary goal of surgical treatment is adequate neural decompression while maintaining spinal stability, alignment, and, when possible, physiological segmental motion.

Anterior cervical discectomy and fusion (ACDF) remains one of the most commonly performed surgical procedures for cervical radiculopathy because it provides reliable anterior decompression and favorable clinical outcomes. However, ACDF is associated with fusion-related and approach-related limitations, including pseudarthrosis, adjacent segment degeneration, implant-related complications, and loss of motion at the treated level [1,2,3,4,5]. These limitations have contributed to increasing interest in posterior motion-preserving decompression techniques for selected patients.

Posterior endoscopic cervical foraminotomy (PECF) has increasingly been used as a minimally invasive motion-preserving procedure for unilateral cervical radiculopathy [1,4,5,6,7,8,9,10,11,12]. The posterior endoscopic approach enables targeted decompression of the exiting nerve root through a small muscle-splitting corridor, with reduced soft-tissue disruption and rapid postoperative recovery. Several studies have reported favorable clinical outcomes after posterior endoscopic and minimally invasive cervical decompression procedures [6,7,8,9,10,11,12,13]. Nevertheless, isolated posterior foraminal decompression may be insufficient in patients with mixed compression patterns, particularly when foraminal stenosis coexists with lateral or foraminal soft disk herniation.

In clinical practice, some patients with cervical radiculopathy present with combined anterior disk-related compression and posterolateral foraminal narrowing caused by hypertrophic bone, ligamentum flavum thickening, or periradicular soft-tissue changes. In such cases, isolated bony decompression or fragment removal alone may not provide complete neural release. Adequate decompression may require addressing both the shoulder and axillary regions of the exiting nerve root and removing compressive structures along the neural corridor.

Combined posterior endoscopic cervical diskectomy and foraminotomy (CEDF) represents a surgical strategy intended to address both foraminal stenosis and concomitant lateral soft disc pathology through a single posterior endoscopic corridor. This approach aims to achieve targeted neural decompression while preserving the facet joint as much as possible and avoiding fusion in carefully selected patients. However, the role of this combined technique relative to established surgical options requires further investigation.

Although clinical outcomes after posterior cervical endoscopic decompression have been reported in previous studies, the relationship between the radiological extent of decompression and postoperative clinical improvement remains insufficiently defined. Most available studies have focused primarily on patient-reported outcomes or qualitative imaging assessment, whereas quantitative evaluation of postoperative foraminal enlargement remains less commonly reported [14,15,16,17,18,19,20,21,22,23,24].

Quantitative magnetic resonance imaging (MRI) may provide a reproducible method for describing postoperative foraminal changes after endoscopic cervical decompression. Parameters such as minimal foraminal diameter, Foraminal Symmetry Index (FSI), and Quantitative Cervical Expansion (QCE) may be useful as preliminary radiological indices for documenting the extent of decompression. However, their clinical validity and relationship with patient-reported outcomes remain uncertain and require further study.

Therefore, the aim of this preliminary retrospective cohort study was to evaluate clinical outcomes and quantitative MRI changes after CEDF in patients with unilateral cervical radiculopathy caused by combined foraminal stenosis and lateral soft disk herniation. Additionally, we explored whether postoperative foraminal enlargement was associated with improvement in arm pain and disability. Given the small sample size and exploratory design, these analyses were considered hypothesis-generating.

## 2. Materials and Methods

### 2.1. Study Design and Ethical Approval

This was a retrospective single-center exploratory cohort study including consecutive patients treated between January 2021 and December 2023 at the University Orthopedic and Rehabilitation Hospital in Zakopane, Poland. The study was designed as a preliminary single-arm analysis of clinical outcomes and quantitative MRI changes after combined posterior endoscopic cervical discectomy and foraminotomy. Given the small sample size and absence of a control group, all outcome and correlation analyses were considered exploratory and hypothesis-generating.

All procedures were performed by the same surgical team using a standardized posterior full-endoscopic cervical technique. The study was conducted in accordance with the Declaration of Helsinki and approved by the Bioethics Committee of the Jagiellonian University Medical College, Kraków, Poland (Approval No. 118.0043.1.463.2025; approval date: 27 November 2025).

### 2.2. Patient Population

Fifteen consecutive patients with unilateral single-level cervical radiculopathy caused by combined foraminal stenosis and lateral or foraminal soft disk herniation were included. The cohort consisted of 9 males and 6 females, with a mean age of 53.8 ± 9.6 years.

All patients presented with clinically confirmed unilateral cervical radiculopathy characterized by radiating arm pain corresponding to the affected nerve root, with or without mild motor weakness in the relevant myotome. Clinical symptoms were consistent with MRI findings at the operated level. All patients had undergone at least 6 weeks of unsuccessful conservative treatment before surgery, including pharmacological treatment, activity modification, and/or physiotherapy.

Inclusion Criteria

- Clinically confirmed unilateral cervical radiculopathy with radiating arm pain corresponding to the affected nerve root, with or without mild motor weakness;
- MRI-confirmed single-level foraminal stenosis with concomitant lateral or foraminal soft disk herniation;
- Failure of at least 6 weeks of conservative treatment;
- Availability of complete preoperative and postoperative MRI studies;
- Minimum clinical follow-up of 12 months.

Exclusion Criteria

- Previous cervical surgery at the index level;
- Cervical myelopathy;
- Multilevel pathology requiring fusion;
- Central canal stenosis;
- Segmental instability on preoperative dynamic flexion–extension radiographs;
- Calcified disk herniation;
- Incomplete clinical or radiological data.

Operated levels included C5–C6 in 6 patients, C6–C7 in 7 patients, and C7–T1 in 2 patients. Baseline demographic and clinical characteristics are summarized in Table 1.

### 2.3. Surgical Technique

All procedures were performed under general anesthesia with patients positioned prone and the head secured using a Mayfield clamp. After fluoroscopic confirmation of the target level, a 7 mm skin incision was made approximately 2–3 cm lateral to the midline. Continuous fluoroscopic guidance was used during docking and decompression to confirm accurate localization and instrument positioning.

Sequential dilators were introduced under fluoroscopic guidance, followed by insertion of a 4.5 mm working-channel endoscope (Richard Wolf GmbH, Knittlingen, Germany). The working cannula was docked at the medial border of the lateral mass.

Under continuous saline irrigation, a high-speed diamond burr was used to perform a limited laminotomy and partial medial facetectomy. Drilling was initiated at the inferior articular process and extended toward the superior articular process to create a keyhole decompression window. Particular attention was paid to preserving more than 50% of the facet joint, in accordance with previously described stability-preserving posterior cervical decompression principles [6,10,13].

After flavectomy, the exiting nerve root and lateral dural margin were identified. Foraminal decompression was continued along the trajectory of the exiting nerve root, including decompression of both the shoulder and axillary regions. Lateral or foraminal soft disk fragments were subsequently removed using endoscopic graspers and pituitary forceps when they were identified as contributing to ventral or axillary nerve root compression.

In this study, combined posterior endoscopic cervical diskectomy and foraminotomy was defined as targeted removal of lateral or foraminal soft disc material combined with posterior foraminal decompression through a single posterior endoscopic corridor. Adequate decompression was assessed intraoperatively by visual confirmation of free mobilization and pulsation of the exiting nerve root and lateral dural margin.

**Figure 1:**
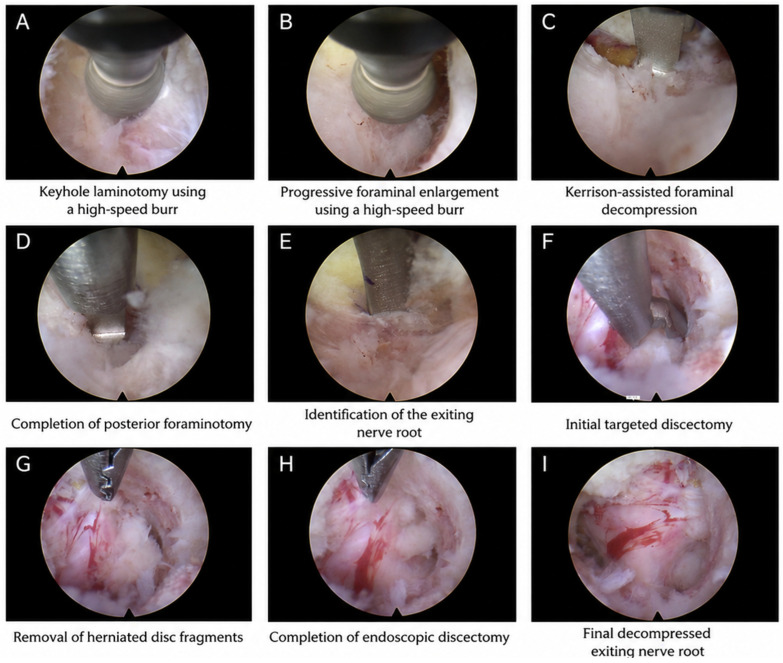
Surgical workflow of combined posterior endoscopic cervical discectomy and foraminotomy. Intraoperative endoscopic views demonstrating the sequential surgical workflow of combined posterior endoscopic cervical discectomy and foraminotomy. (**A**,**B**) Keyhole laminotomy and progressive foraminal enlargement performed using a high-speed diamond burr. (**C**,**D**) Completion of posterior foraminotomy and decompression using Kerrison punches while preserving the facet joint. (**E**) Identification and exposure of the exiting cervical nerve root after foraminal decompression. (**F**–**H**) Targeted endoscopic removal of lateral or foraminal soft disc fragments from the axillary and ventral aspect of the nerve root. (**I**) Final view demonstrating free mobilization and decompression of the exiting nerve root.

The sequential surgical workflow of CEDF is illustrated in Figure 1.

Hemostasis was achieved using bipolar radiofrequency coagulation. No drains were used. Patients were mobilized within several hours after surgery and discharged within 24 h when neurological status was stable and no immediate postoperative complications were observed.

Perioperative and postoperative adverse events were recorded, including neurological deterioration, cerebrospinal fluid leak, wound infection, postoperative hematoma, readmission, revision surgery, and radiographic instability during follow-up.

### 2.4. Radiological Assessment

Magnetic resonance imaging (MRI) examinations were performed preoperatively and between 3 and 6 months postoperatively using a 1.5-T scanner (Siemens Avanto Fit, Siemens Healthineers, Erlangen, Germany). Standard clinical MRI sequences were reviewed at the index level to confirm the operated side, the presence of foraminal stenosis with concomitant lateral or foraminal soft disk herniation, and the presence or absence of residual postoperative nerve root compression.

Measurements were performed using K-PACS DICOM software version 1.6.0 (Image Information Systems Ltd., Rostock, Germany). Axial T2-weighted images were reviewed together with corresponding sagittal and coronal sequences, when available, to identify the narrowest reproducible foraminal segment at the operated level. The minimal foraminal diameter was measured at the narrowest point of the neural foramen along the apparent trajectory of the exiting nerve root. Measurements were matched preoperatively and postoperatively according to the operated level, nerve root trajectory, and site of maximal preoperative stenosis.

The symptomatic foramen and contralateral foramen were measured at analogous anatomical points at the same index level. Postoperative measurements were obtained at the corresponding location to allow within-patient comparison of foraminal caliber before and after decompression. In cases where postoperative soft-tissue remodeling, scar tissue, or partial volume effects were present, the narrowest reproducible point corresponding to the preoperative measurement site was selected.

Because the MRI protocol was based on standard two-dimensional clinical sequences, this measurement was not intended to represent a true three-dimensional volumetric assessment of the cervical neural foramen, but rather a standardized within-patient index of foraminal caliber for preoperative and postoperative comparison. MRI measurements were adapted from previously published cervical foraminal stenosis assessment methods [15,17,18,19,20,21,22,23,24].

To improve reproducibility, all measurements were independently performed by two fellowship-trained spine surgeons who were not involved in postoperative clinical evaluation and were blinded to clinical outcomes. Each observer repeated all measurements after a 4-week interval. The mean value of the four measurements was used for final analysis.

The following quantitative radiological parameters were analyzed:

- ΔForamen (mm) = postoperative foraminal diameter − preoperative foraminal diameter;
- Foraminal Symmetry Index (FSI) = symptomatic side diameter/contralateral side diameter;
- Quantitative Cervical Expansion (QCE) = (ΔForamen/preoperative diameter) × 100%.

Interobserver and intraobserver reliability were evaluated using intraclass correlation coefficients (ICC). These measurements were considered exploratory quantitative radiological indices rather than validated biomarkers of decompression adequacy or clinical response.

### 2.5. Clinical Evaluation

Clinical outcomes were assessed preoperatively, at 6 weeks, and at 12 months postoperatively. Patient-reported outcome measures included the Visual Analog Scale for arm pain (VAS-arm), the Visual Analog Scale for neck pain (VAS-neck), the Neck Disability Index (NDI), and the modified MacNab criteria.

Preoperative and postoperative neurological status was assessed clinically, including motor strength, sensory disturbance, and reflex abnormalities when present. Mild motor weakness was defined as a decrease in motor strength in the relevant myotome without severe or progressive neurological deficit.

The minimum clinically important difference (MCID) for VAS-arm improvement was defined as a reduction of ≥2 points, based on commonly used thresholds for clinically meaningful pain reduction in clinical pain studies [25]. Perioperative and postoperative adverse events were recorded, including neurological deterioration, cerebrospinal fluid leak, wound infection, postoperative hematoma, hospital readmission, and revision surgery.

Segmental stability was assessed in all patients using dynamic flexion–extension radiographs obtained preoperatively and during postoperative follow-up. Radiographic instability was defined as abnormal segmental translation or excessive angular motion at the operated level on flexion–extension radiographs, as assessed by the treating spine surgeons.

Clinical outcome assessment followed commonly used criteria in cervical endoscopic spine surgery studies [4,5,6,9,10,11].

### 2.6. Statistical Analysis

Statistical analysis was performed using IBM SPSS Statistics version 27 (IBM Corp., Armonk, NY, USA). Continuous variables were presented as mean ± standard deviation (SD), and categorical variables were presented as number of patients and percentage. Normality of distribution was assessed using the Shapiro–Wilk test.

Preoperative and postoperative values were compared using paired Student’s *t*-tests or Wilcoxon signed-rank tests, as appropriate. Correlations between radiological and clinical outcomes, including ΔForamen, FSI, QCE, ΔVAS-arm, and ΔNDI, were analyzed using Pearson correlation coefficients for normally distributed variables and Spearman rank correlation analysis for nonnormally distributed variables.

Given the small sample size and retrospective single-arm design, all statistical analyses were considered exploratory and hypothesis-generating. No formal sample size calculation was performed. Correlation analyses were interpreted cautiously and were not intended to establish predictive validity of the radiological parameters.

Interobserver and intraobserver agreement were evaluated using intraclass correlation coefficients (ICC). A *p*-value < 0.05 was considered statistically significant.

## 3. Results

### 3.1. Patient Characteristics

A total of 15 consecutive patients met the inclusion criteria and completed the minimum 12-month follow-up. No patients were lost to follow-up. The study cohort consisted of 9 males and 6 females, with a mean age of 53.8 ± 9.6 years and a mean symptom duration of 8.2 ± 4.7 months.

All patients presented with clinically confirmed unilateral cervical radiculopathy characterized by radiating arm pain corresponding to the affected nerve root and MRI-confirmed single-level foraminal stenosis with concomitant lateral or foraminal soft disk herniation. Mild motor weakness was present preoperatively in 4 patients (26.7%). All patients had undergone at least 6 weeks of unsuccessful conservative treatment before surgery.

The most commonly treated level was C6–C7 in 7 patients (46.7%), followed by C5–C6 in 6 patients (40.0%) and C7–T1 in 2 patients (13.3%). The mean follow-up duration was 14.6 ± 2.8 months. Baseline demographic and clinical characteristics are summarized in Table 1.

### 3.2. Clinical Outcomes

Postoperative improvement was observed across all evaluated clinical outcome measures (Table 2).

Mean VAS-arm decreased from 7.2 ± 1.3 preoperatively to 2.1 ± 1.5 at 6 weeks and 1.3 ± 1.4 at 12 months. Mean VAS-neck decreased from 4.8 ± 1.7 preoperatively to 2.7 ± 1.4 at 6 weeks and 1.9 ± 1.2 at 12 months.

Functional disability also improved during follow-up. Mean NDI decreased from 48.0 ± 14.0% preoperatively to 24.6 ± 11.3% at 6 weeks and 18.3 ± 12.0% at 12 months.

The minimum clinically important difference for arm pain reduction was achieved in 13 of 15 patients (86.7%) at 6 weeks and in 14 of 15 patients (93.3%) at 12 months. According to the modified MacNab criteria, excellent or good outcomes were observed in 12 patients (80.0%) at 6 weeks and in 14 patients (93.3%) at final follow-up.

**Figure 2:**
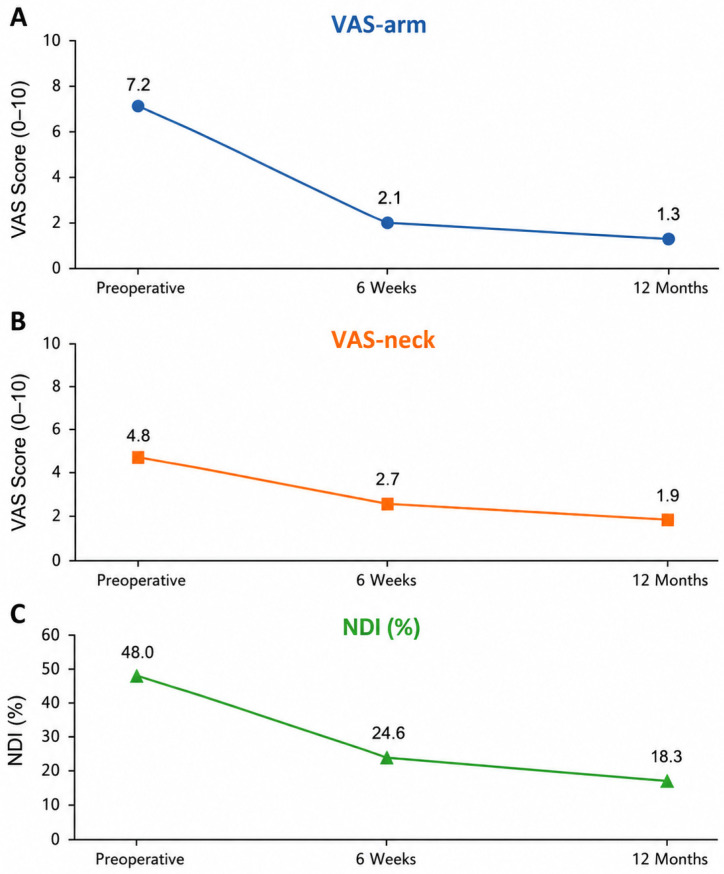
Clinical outcomes after combined posterior endoscopic cervical discectomy and foraminotomy. Postoperative changes in patient-reported clinical outcome measures are shown separately for each scale. (**A**) Mean Visual Analog Scale for arm pain (VAS-arm) decreased from 7.2 preoperatively to 2.1 at 6 weeks and 1.3 at 12 months. (**B**) Mean Visual Analog Scale for neck pain (VAS-neck) decreased from 4.8 preoperatively to 2.7 at 6 weeks and 1.9 at 12 months. (**C**) Mean Neck Disability Index (NDI) decreased from 48.0% preoperatively to 24.6% at 6 weeks and 18.3% at 12 months. NDI—Neck Disability Index; VAS—Visual Analog Scale.

Mild preoperative motor weakness was present in 4 patients (26.7%). Postoperative improvement of motor strength was observed in all affected patients during follow-up. No postoperative neurological deterioration was recorded. No revision procedures, postoperative instability, deep infections, cerebrospinal fluid leaks, or hospital readmissions occurred during the available follow-up period. The observed improvements in pain and functional outcomes are illustrated in Figure 2.

### 3.3. Radiological Outcomes

Quantitative MRI assessment demonstrated postoperative enlargement of the symptomatic neural foramen and improvement in foraminal symmetry (Table 3).

Mean minimal foraminal diameter increased from 1.9 ± 0.7 mm preoperatively to 4.1 ± 0.8 mm postoperatively, corresponding to a mean increase of 2.2 mm. The Foraminal Symmetry Index improved from 0.40 ± 0.12 preoperatively to 0.89 ± 0.11 postoperatively, indicating improved symmetry between the symptomatic and contralateral foramina after decompression.

Mean Quantitative Cervical Expansion was 121.5 ± 38.4%, reflecting measurable postoperative enlargement of the symptomatic neural foramen. Interobserver and intraobserver reliability analyses demonstrated high measurement reproducibility, with ICC values of 0.91 and 0.93, respectively.

**Figure 3:**
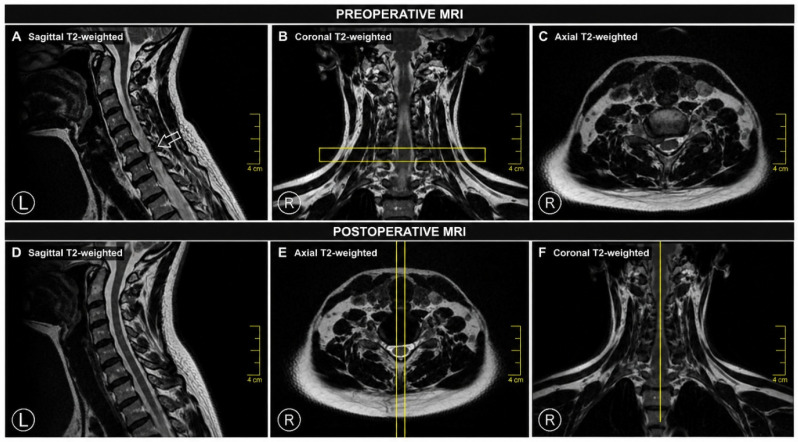
Representative preoperative and postoperative MRI findings after combined posterior endoscopic cervical diskectomy and foraminotomy. (**A**–**C**) Preoperative sagittal, coronal, and axial T2-weighted MRI images demonstrating unilateral cervical foraminal stenosis with concomitant lateral soft disc herniation and compression of the exiting cervical nerve root. The white arrow in panel A indicates the lateral soft disc herniation at the C6/C7 level, and the yellow box in panel B indicates the corresponding C6/C7 disc level in the coronal plane. (**D**–**F**) Postoperative sagittal, axial, and coronal T2-weighted MRI images obtained after combined posterior endoscopic cervical discectomy and foraminotomy, demonstrating increased foraminal caliber and visible decompression of the exiting nerve root, with no evident residual nerve root compression on postoperative MRI. The yellow midline reference markers in panels E and F indicate the median anatomical plane and serve as orientation landmarks for postoperative imaging assessment. MRI—magnetic resonance imaging.

Representative preoperative and postoperative MRI findings are presented in Figure 3.

### 3.4. Correlation Between Radiological and Clinical Outcomes

Exploratory correlation analysis was performed to evaluate the relationship between quantitative MRI parameters and clinical improvement. Greater postoperative foraminal enlargement showed a moderate positive association with reduction in arm pain severity, with larger ΔForamen values associated with greater reductions in VAS-arm scores (r = 0.54, *p* = 0.045).

Higher QCE values also showed a positive trend toward greater arm pain reduction; however, this association did not reach statistical significance. No significant correlation was identified between postoperative foraminal enlargement and improvement in VAS-neck scores.

Given the small sample size and exploratory design, these correlation findings should be interpreted cautiously and should not be considered evidence of predictive validity. Rather, they represent preliminary hypothesis-generating observations suggesting that quantitative MRI-based foraminal measurements may be useful in future studies evaluating radiological decompression after endoscopic cervical surgery.

**Figure 4:**
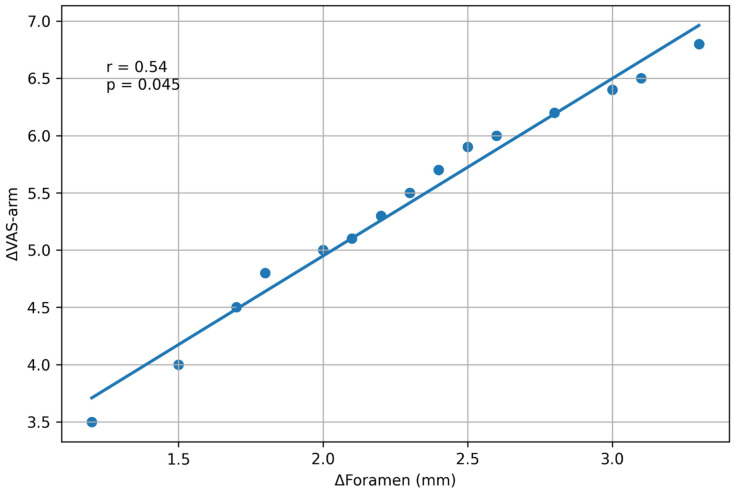
Exploratory association between postoperative foraminal enlargement and reduction in arm pain after combined posterior endoscopic cervical discectomy and foraminotomy.

The relationship between postoperative foraminal enlargement and reduction in arm pain severity is illustrated in Figure 4.

Scatter plot showing the relationship between postoperative foraminal enlargement (ΔForamen) and reduction in arm pain severity (ΔVAS-arm). A moderate positive exploratory association was observed between greater foraminal enlargement and greater reduction in arm pain scores (r = 0.54, *p* = 0.045). The solid line represents the linear regression line with 95% confidence intervals. Given the small sample size, this association should be interpreted cautiously and considered hypothesis-generating. ΔVAS-arm represents the difference between preoperative and postoperative VAS-arm scores; CEDF—combined endoscopic discectomy and foraminotomy; VAS—Visual Analog Scale.

## 4. Discussion

### 4.1. Main Findings

The main finding of this preliminary retrospective cohort study was that combined posterior endoscopic cervical discectomy and foraminotomy was associated with postoperative improvement in pain and disability, together with measurable enlargement of the symptomatic neural foramen on quantitative MRI assessment. In addition, an exploratory moderate positive association was observed between postoperative foraminal enlargement and reduction in arm pain severity.

Patients demonstrated improvement in arm pain, neck pain, and functional disability during follow-up, with 14 of 15 patients achieving the minimum clinically important difference for arm pain reduction at 12 months. Quantitative MRI assessment showed an increase in minimal foraminal diameter and improvement in foraminal symmetry after decompression. These findings suggest that combined decompression of foraminal stenosis and concomitant lateral or foraminal soft disk pathology may be clinically relevant in carefully selected patients.

However, the observed association between radiological decompression and clinical improvement should be interpreted cautiously because of the small sample size, retrospective design, and absence of a control group. Therefore, the present results should be regarded as preliminary and hypothesis-generating rather than confirmatory evidence that quantitative MRI parameters can predict clinical response.

No postoperative neurological deterioration, revision procedures, or radiographic signs of instability were observed during the available follow-up. Although this observation is reassuring, the cohort is too small to draw definitive conclusions regarding the safety profile or long-term durability of the combined posterior endoscopic approach.

### 4.2. Clinical Significance of Combined Decompression

Posterior cervical foraminotomy, including minimally invasive and endoscopic techniques, has increasingly been used for selected patients with unilateral cervical radiculopathy, particularly when the pathology is predominantly foraminal or posterolateral [1,4,5,6,7,8,9,10,11,12]. Compared with anterior fusion-based procedures, posterior decompression has the theoretical advantage of preserving the motion segment and avoiding anterior approach-related morbidity; however, the choice of surgical approach depends on the location and type of neural compression, cervical alignment, segmental stability, and surgeon experience [1,2,3,4,5,9,10,11,12]. Ongoing comparative research has further highlighted the need to define the relative role of posterior and anterior decompression strategies for cervical foraminal stenosis [26].

A clinically relevant challenge occurs when foraminal stenosis coexists with lateral or foraminal soft disk herniation. In this mixed compression pattern, isolated bony foraminal decompression may not fully address ventral or axillary nerve root compression, whereas isolated fragment removal may not sufficiently enlarge the narrowed foramen. Therefore, selected patients may require decompression of both the foraminal and ventral components of nerve root compression.

In the present study, CEDF was used as a combined posterior endoscopic strategy integrating targeted removal of lateral or foraminal soft disk material with posterior foraminal decompression through a single endoscopic corridor. The goal of this approach was to decompress both the shoulder and axillary regions of the exiting nerve root while preserving more than 50% of the facet joint, in accordance with stability-preserving principles described for posterior cervical decompression [6,10,13].

The clinical improvement observed in this cohort suggests that addressing both foraminal stenosis and concomitant lateral soft disk pathology may be beneficial in carefully selected patients with unilateral cervical radiculopathy. However, because this study did not include a control group, it cannot determine whether combined decompression provides superior, equivalent, or distinct benefit compared with isolated posterior foraminotomy, conventional posterior cervical foraminotomy, or ACDF.

No postoperative instability or revision procedures were observed during the available follow-up. Nevertheless, this observation should be interpreted cautiously because of the small cohort size and relatively short follow-up. Larger prospective comparative studies are required to define the indications, safety profile, and relative clinical value of CEDF among established cervical decompression procedures.

### 4.3. Quantitative MRI Assessment and Clinical Correlation

One of the aims of the present study was to explore whether quantitative MRI-derived measurements could be used to describe postoperative foraminal changes after combined posterior endoscopic cervical decompression. Although several previous studies have assessed clinical outcomes after posterior cervical decompression, fewer investigations have attempted to quantify postoperative foraminal enlargement and relate imaging findings to symptom improvement [14,15,16,17,18,19,20,21,22,23,24]. Quantitative postoperative assessment has also been explored in endoscopic foraminal decompression outside the cervical spine, supporting the broader relevance of imaging-based evaluation after minimally invasive foraminal surgery [27].

In this cohort, quantitative MRI assessment demonstrated an increase in minimal foraminal diameter and improvement in foraminal symmetry after decompression. An exploratory moderate positive association was observed between postoperative foraminal enlargement and reduction in arm pain severity. However, because of the small sample size and single-arm retrospective design, this association should be interpreted cautiously and should not be considered evidence that MRI-derived measurements can predict clinical response.

The proposed parameters, including the Foraminal Symmetry Index (FSI) and Quantitative Cervical Expansion (QCE), demonstrated high interobserver and intraobserver reproducibility in this cohort. This suggests that these measurements may be feasible for standardized radiological reporting in future studies. However, reproducibility does not establish clinical validity. Therefore, FSI and QCE should be regarded as preliminary quantitative radiological indices rather than validated biomarkers of decompression adequacy or postoperative outcome.

The use of quantitative imaging assessment may be particularly relevant in minimally invasive spine surgery, where the extent of decompression is achieved through limited operative corridors and may be difficult to evaluate using qualitative imaging alone. Nevertheless, postoperative symptom improvement is multifactorial and may depend on neural recovery, inflammatory changes, duration of preoperative compression, baseline neurological status, psychosocial factors, and rehabilitation. Therefore, radiological enlargement of the neural foramen should not be interpreted as the sole determinant of clinical recovery.

Future prospective studies should validate these measurements in larger cohorts, ideally using standardized MRI protocols, oblique sagittal sequences, three-dimensional imaging, or CT-based morphometric assessment. Comparative studies are also needed to determine whether quantitative foraminal measurements add clinically meaningful information beyond conventional patient-reported outcomes and qualitative postoperative imaging.

### 4.4. Comparison with Previous Literature

The clinical outcomes observed in the present cohort are generally consistent with previously published studies on posterior endoscopic and minimally invasive cervical decompression. Ruetten et al. reported favorable outcomes after full-endoscopic posterior cervical foraminotomy for lateral cervical disk herniation, while subsequent studies and systematic reviews have described satisfactory symptom relief after posterior endoscopic cervical decompression in selected patients with unilateral cervical radiculopathy [1,4,5,6,7,8,9,10,11,12].

Larger clinical series, including the study by Zheng et al., have demonstrated that posterior percutaneous endoscopic cervical decompression can be performed in a broad range of patients, with acceptable complication and revision profiles in experienced hands [9]. Comparative studies and systematic reviews have also suggested that posterior cervical foraminotomy may provide clinical outcomes comparable to ACDF in appropriately selected patients while avoiding fusion-related limitations and anterior approach-related morbidity [1,2,4,5]. However, these findings should not be directly extrapolated to the present cohort because our study was small, retrospective, and did not include a control group.

Most previous investigations have focused primarily on patient-reported outcomes and qualitative radiological evaluation. Quantitative assessment of postoperative foraminal enlargement remains less frequently reported in the cervical spine literature. Mizouchi et al. analyzed radiographic restenosis after posterior cervical foraminotomy and emphasized the relevance of postoperative foraminal evaluation during follow-up [14]. In addition, several MRI- and CT-based grading systems or morphometric methods have been proposed for assessing cervical foraminal stenosis [15,17,18,19,20,21,22,23,24].

The present study adds preliminary data by combining patient-reported clinical outcomes with quantitative MRI-based assessment of postoperative foraminal changes after CEDF. The exploratory association between foraminal enlargement and arm pain reduction may support further investigation of quantitative radiological indices in future studies. However, given the small sample size and borderline statistical significance, this finding should be interpreted as hypothesis-generating rather than confirmatory.

Therefore, the current results should be viewed as an early contribution to the evolving literature on quantitative postoperative imaging after endoscopic cervical decompression. Larger prospective studies with standardized imaging protocols and comparative control groups are needed to determine whether MRI-derived foraminal measurements provide clinically meaningful information beyond conventional clinical and qualitative radiological assessment.

### 4.5. Motion Preservation and Minimally Invasive Advantages

Motion preservation is one of the theoretical advantages of posterior cervical decompression compared with fusion-based anterior procedures. By avoiding interbody fusion, posterior endoscopic decompression may preserve segmental motion and avoid implant-related complications or anterior approach-related morbidity in appropriately selected patients [1,2,3,5,7]. However, the present study did not include dynamic motion analysis or a comparative ACDF group; therefore, no conclusions can be drawn regarding the long-term biomechanical or motion-preserving advantages of CEDF.

The posterior endoscopic approach used in this study was performed through a small muscle-splitting corridor and was intended to minimize paraspinal soft-tissue disruption. In this cohort, all patients were mobilized within several hours after surgery and discharged within 24 h, suggesting that the procedure was feasible in a short-stay surgical setting. Nevertheless, the small sample size prevents any definitive conclusions regarding perioperative morbidity, recovery speed, or safety.

The ability to address both foraminal stenosis and lateral or foraminal soft disk pathology through a single posterior endoscopic corridor may be clinically relevant in selected patients. However, whether this combined approach expands the indications for motion-preserving cervical surgery remains uncertain and should be evaluated in larger prospective comparative studies.

### 4.6. Limitations

Several important limitations of the present study should be acknowledged. First, this was a retrospective single-center study including a very small cohort of 15 patients. This substantially limits statistical power, reproducibility, and generalizability of the findings. Therefore, all outcome analyses and correlation analyses should be interpreted as exploratory and hypothesis-generating rather than confirmatory.

Second, the absence of a control group precludes direct comparison with isolated posterior endoscopic foraminotomy, conventional posterior cervical foraminotomy, or ACDF. As a result, this study cannot determine whether CEDF provides superior, equivalent, or distinct clinical benefit compared with established surgical alternatives.

Third, although no major neurological complications, revision procedures, or radiographic instability were observed in this cohort, the sample size is insufficient to draw definitive conclusions regarding the safety profile of the technique. Rare but clinically important complications would not be expected to be detected in such a small cohort.

Fourth, the mean follow-up duration was relatively short, and postoperative MRI examinations were performed between 3 and 6 months after surgery. Therefore, longer-term radiological remodeling, recurrent foraminal narrowing, delayed instability, and durability of clinical improvement could not be assessed.

Fifth, the radiological assessment was based on standard clinical MRI sequences. Although axial T2-weighted images were reviewed together with corresponding sagittal and coronal sequences when available, the cervical neural foramen has an oblique and three-dimensional morphology. Therefore, the minimal foraminal diameter used in this study should be regarded as a standardized within-patient index of foraminal caliber rather than a true three-dimensional volumetric measurement. Future studies should incorporate standardized oblique sagittal MRI, three-dimensional MRI sequences, or CT-based morphometric assessment.

Sixth, although the proposed quantitative MRI parameters demonstrated high interobserver and intraobserver reproducibility, reproducibility does not establish clinical validity. FSI and QCE should therefore be considered preliminary quantitative radiological indices rather than validated biomarkers of decompression adequacy or clinical response.

Finally, the clinical assessment was limited to VAS-arm, VAS-neck, NDI, modified MacNab criteria, neurological status, and adverse events. More detailed clinical information, including analgesic use, return to work or activity, patient satisfaction, and standardized health-related quality-of-life measures, was not available for all patients because of the retrospective study design. Postoperative recovery is likely multifactorial and may be influenced by neurological recovery, inflammatory changes, symptom duration, psychosocial factors, and rehabilitation, in addition to foraminal dimensions alone.

Despite these limitations, the present study provides preliminary data on clinical outcomes and quantitative MRI-based assessment after CEDF. The findings may help inform the design of future prospective comparative studies evaluating standardized radiological outcome measures after endoscopic cervical decompression.

## 5. Conclusions

In this preliminary retrospective single-center cohort study, combined posterior endoscopic cervical diskectomy and foraminotomy was associated with postoperative improvement in pain and disability, together with measurable enlargement of the symptomatic neural foramen in carefully selected patients with unilateral cervical radiculopathy caused by combined foraminal stenosis and lateral or foraminal soft disc herniation.

Quantitative MRI assessment demonstrated increased minimal foraminal diameter and improved foraminal symmetry after decompression. An exploratory association was observed between postoperative foraminal enlargement and reduction in arm pain severity; however, this finding should be interpreted cautiously because of the small sample size, retrospective design, and absence of a control group.

The proposed MRI-derived parameters, including the Foraminal Symmetry Index and Quantitative Cervical Expansion, demonstrated high measurement reproducibility in this cohort. However, they should be regarded as preliminary quantitative radiological indices rather than validated markers of decompression adequacy or clinical response.

No major neurological complications, revision procedures, or radiographic instability were observed during the available follow-up. Nevertheless, this study is underpowered to draw definitive conclusions regarding safety, long-term durability, or comparative effectiveness.

Further prospective multicenter studies with larger patient cohorts, longer follow-up, standardized imaging protocols, and appropriate control groups are required to validate these imaging-based measurements and define the role of combined posterior endoscopic cervical discectomy and foraminotomy among established cervical decompression procedures.

## Figures and Tables

**Table 1:** Baseline demographic and clinical characteristics of the study population.

Characteristic	Value
Number of patients	15
Age, years	53.8 ± 9.6
Sex, male/female	9/6
Symptom duration, months	8.2 ± 4.7
Side of symptoms, right/left	8/7
Operated level	
C5–C6	6 (40.0%)
C6–C7	7 (46.7%)
C7–T1	2 (13.3%)
Foraminal stenosis with concomitant soft disk herniation	15 (100%)
Clinically confirmed unilateral cervical radiculopathy	15 (100%)
Radiating arm pain corresponding to the affected nerve root	15 (100%)
Mild motor weakness	4/15 (26.7%)
Failed conservative treatment ≥ 6 weeks	15 (100%)
Preoperative VAS-arm	7.2 ± 1.3
Preoperative VAS-neck	4.8 ± 1.7
Preoperative NDI, %	48.0 ± 14.0
Minimum follow-up, months	12
Mean follow-up, months	14.6 ± 2.8

Data are presented as mean ± standard deviation or number of patients (%). NDI—Neck Disability Index; VAS—Visual Analog Scale.

**Table 2:** Clinical outcomes after combined posterior endoscopic cervical discectomy and foraminotomy.

Outcome Measure	Preoperative	6 Weeks	12 Months	*p*-Value
VAS-arm	7.2 ± 1.3	2.1 ± 1.5	1.3 ± 1.4	<0.001
VAS-neck	4.8 ± 1.7	2.7 ± 1.4	1.9 ± 1.2	<0.001
NDI, %	48.0 ± 14.0	24.6 ± 11.3	18.3 ± 12.0	<0.001
Patients achieving MCID for VAS-arm	—	13/15 (86.7%)	14/15 (93.3%)	—
Excellent/good MacNab outcome	—	12/15 (80.0%)	14/15 (93.3%)	—

Values are presented as mean ± standard deviation or number of patients (%), unless otherwise indicated. MCID—minimum clinically important difference; NDI—Neck Disability Index; VAS—Visual Analog Scale.

**Table 3:** Quantitative MRI parameters before and after surgery.

Radiological Parameter	Preoperative	Postoperative	Change	*p*-Value
Minimal foraminal diameter, mm	1.9 ± 0.7	4.1 ± 0.8	+2.2 mm	<0.001
Foraminal Symmetry Index	0.40 ± 0.12	0.89 ± 0.11	+0.49	<0.001
Quantitative Cervical Expansion, %	—	121.5 ± 38.4	—	—
Interobserver ICC	—	0.91	—	—
Intraobserver ICC	—	0.93	—	—

Values are presented as mean ± standard deviation, unless otherwise indicated. ICC—intraclass correlation coefficient.

## Data Availability

Data presented in this study are available on reasonable request from the corresponding author. Data are not publicly available due to privacy and ethical restrictions.

## Abbreviations

ACDF	Anterior Cervical Discectomy and Fusion
CEDF	Combined Endoscopic Discectomy and Foraminotomy
FSI	Foraminal Symmetry Index
ICC	Intraclass Correlation Coefficient
MCID	Minimum Clinically Important Difference
MRI	Magnetic Resonance Imaging
NDI	Neck Disability Index
PECF	Posterior Endoscopic Cervical Foraminotomy
QCE	Quantitative Cervical Expansion
SD	Standard Deviation
VAS	Visual Analog Scale
VAS-arm	Visual Analog Scale for Arm Pain
VAS-neck	Visual Analog Scale for Neck Pain
